# Broadband and enhanced nonlinear optical response of MoS_2_/graphene nanocomposites for ultrafast photonics applications

**DOI:** 10.1038/srep16372

**Published:** 2015-11-09

**Authors:** Yaqin Jiang, Lili Miao, Guobao Jiang, Yu Chen, Xiang Qi, Xiao-fang Jiang, Han Zhang, Shuangchun Wen

**Affiliations:** 1 Key Laboratory for Micro-/Nano-Optoelectronic Devices of Ministry of Education, School of Physics and Electronics, Hunan University, Changsha 410082, China; 2 SZU-NUS Collaborative Innovation Center for Optoelectronic Science & Technology, Shenzhen University, Shenzhen 518060, China; 3 Key Laboratory of Optoelectronic Devices and Systems of Ministry of Education and Guangdong Province, Shenzhen University, Shenzhen 518060, China; 4 Hunan Provincial Key Laboratory of Micro-Nano Energy Materials and Devices, Laboratory for Quantum Engineering and Micro-Nano Energy Technology, Xiangtan University, Hunan 411105, China; 5 State Key Laboratory of luminescent Materials and Devices, South China University of Technology, Guangzhou 510000, China

## Abstract

Due to their relatively high compatibility with specific photonic structures, strong light-matter interactions and unique nonlinear optical response, two-dimensional (2D) materials, such as graphene and transition metal dichalcogenides, are attractive for ultrafast photonics applications. Here, we fabricate MoS_2_/graphene nanocomposites by a typical hydrothermal method. In addition, we systematically investigate their nonlinear optical responses. Our experiments indicate that the combined advantages of ultrafast relaxation, a broadband response from graphene, and the strong light-matter interaction from MoS_2_, can be integrated together by composition. The optical properties in terms of carrier relaxation dynamics, saturation intensity and modulation depth suggest great potential for the MoS_2_/graphene nanocomposites in photonics applications. We have further fabricated 2D nanocomposites based optical saturable absorbers and integrated them into a 1.5 μm Erbium-doped fiber laser to demonstrate Q-switched and mode-locked pulse generation. The fabrication of 2D nanocomposites assembled from different types of 2D materials, via this simple and scalable growth approach, paves the way for the formation and tuning of new 2D materials with desirable photonic properties and applications.

Two-dimensional (2D) materials have received increasing attention in recent years because of their unique optoelectronic properties, which are a natural consequence of the quantum confinement effect associated with their ultra-thin 2D structure^1^,^2^,^3^. Graphene, a layer of 2D sp^2^-bonded carbon atoms, has been widely researched for optoelectronic applications due to its high carrier mobility^4^,^5^, broad absorption spectrum^6^, remarkable nonlinear optical (NLO) properties^6^,^7^ and ultrafast carrier dynamics^8^,^9^,^10^,^11^. Enlightened by the substantial advantages of graphene for optoelectronics, researchers have started to explore the graphene analogues of layered inorganic materials. Another type of 2D materials are transition metal dichalcogenides (TMDCs), such as MoS_2_, MoSe_2_, WS_2_ and WSe_2_, consisting of a hexagonal layer of metal atoms (M) sandwiched between two layers of chalcogen atoms (X) within the stoichiometry MX_2_^12^. Their common characteristic is a layered structure with strong intra-layer covalent bonding and weak interlayer Van der Waals forces between the MX_2_ sheets. Single- and few-layer 2D TMDCs materials have been successfully fabricated through different approaches, such as mechanical or liquid exfoliation^12^,^13^,^14^,^15^. In terms of their applications, TMDCs have already exhibited considerable potentials in various fields including field-effect transistors^16^,^17^,^18^,^19^, photodetectors^20^, memories^21^, integrated circuits^22^,^23^, passive mode lockers^24^,^25^ and optical switchers^26^. Among them, molybdenum disulphide (MoS_2_) has been intensively studied. Unlike the semi-metallic graphene, MoS_2_ is a semiconductor with a band-gap that is dependent on the number of layers and varies from 1.29 eV^1^ (bulk) to 1.9 eV (single layer)^27^,^28^. The band structure of MoS_2_ has an indirect-to-direct transition when the thickness is reduced from bulk to monolayer^1^,^28^, resulting in the dramatic enhancement of photoluminescence^28^. Otherwise, MoS_2_ shows strong light-matter interaction because of Van Hove singularities in the density of states^29^. The exotic optical-electrical properties make MoS_2_ a potential optical material for use in optoelectronics. Most recently, the fabrication of 2D heterostructures or nanocomposites assembled by graphene and other graphene-like 2D crystals (such as MoS_2_) has been demonstrated as a useful strategy for the realization of novel 2D materials with unique electronic and optoelectronic applications^30^,^31^. The strongest motivation to investigate the nanocomposites is to determine their multiple functionalities. Therefore, to further enhance the optical properties of graphene as well as those of the newly introduced functional materials, it is necessary to investigate the possibility of combining graphene and other 2D materials by specific methods to create 2D nanocomposites. Recently, MoS_2_/graphene nanocomposites were successfully synthesized^32^,^33^, and they show good electron conductivity in electrochemical applications. Interestingly, MoS_2_/graphene nanocomposites have also been reported for various applications of dye-sensitive solar cell fabrication^34^, reversible lithium storage^32^,^35^, novel biosensors and electronic devices^36^. MoS_2_/graphene nanocomposites may have similar properties and comparable performance as materials containing composites^37^. Although experimentalists have shown great interests in other properties of MoS_2_/graphene nanocomposites, their ultrafast carrier dynamics and nonlinear optical properties remain un-explored. However, understanding the ultrafast and nonlinear optical properties is critical for the use of MoS_2_/graphene nanocomposites in ultrafast photonics applications. Herein, we have investigated the carrier dynamics of the photo-excited states of MoS_2_/graphene nanocomposites by ultrafast pump-probe measurement with pump and probe wavelength in the near-infrared region (800 nm). Our results indicate that the carrier relaxation time of the MoS_2_/graphene nanocomposites is longer than that of graphene due to the existence of a covalent donor–acceptor structure between MoS_2_ and graphene. We have further studied the nonlinear absorption through the open aperture Z-scan system from the visible (400 nm) to the near-infrared (1560 nm) broadband frequency range. These findings suggest that MoS_2_/graphene nanocomposites could potentially be used as a passive Q-switcher and mode-locker in ultrafast lasers and ultrafast optical switches.

Inspired by the excellent performance of graphene based saturable absorbers (SAs), many other graphene-like 2D nanomaterials, such as topological insulators (TIs, for example, Bi_2_Se_3_^38^, Bi_2_Te_3_^39^ and Sb_2_Te_3_^40^,^41^), and transition metal dichalcogenides (TMDs, for example, MoS_2_^24^,^42^), have also been widely considered as candidate materials for new types of SAs. The use of these materials has led to the generation of ultrashort pulse with improved performance.

MoS_2_/graphene nanocomposites based optical SAs were fabricated and introduced into an erbium-doped optical fiber laser setup, which could deliver either a passively Q-switched or mode-locked fiber laser operation. The superiority of MoS_2_/graphene nanocomposites will facilitate potential applications of 2D materials for ultrafast photonics, such as Q-switcher, passive laser mode locker, and optical switcher.

## Results

### Characterizations of MoS_2_/graphene nanocomposites

**Figure 1:**
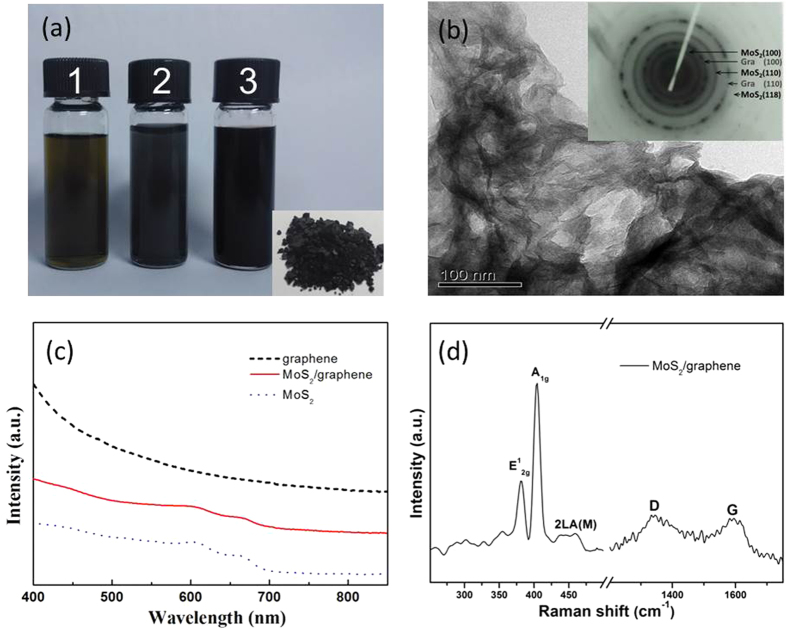
Characterizations of the MoS_2_/graphene nanocomposites. (**a**) Photographs of the MoS_2_ solution (1), graphene (2) and the MoS_2_/graphene nanocomposites (3) dispersed in NMP, insert: the black solid MoS_2_/graphene nanocomposites. (**b**) TEM image of the MoS_2_/graphene nanocomposites (inset: selected area electron diffraction (SEAD) pattern of MoS_2_/graphene nanocomposites). (**c**) UV-Vis spectra of graphene (black dashed line), MoS_2_/graphene nanocomposites (red solid line) and MoS_2_ (blue dotted line). (**d**) Raman spectrum of the MoS_2_/graphene nanocomposites.

The characterization of MoS_2_/graphene nanocomposites samples is summarized in Fig. 1. Figure 1(a) shows photographs of the MoS_2_ solution (1), graphene (2) and MoS_2_/graphene nanocomposites (3) dispersed uniformly in N-methyl-2-pyrrolidone (NMP), whereas the inset shows the black solid MoS_2_/graphene nanocomposites. The transmission electron microscope (TEM) image of MoS_2_/graphene nanocomposites (Fig. 1(b)) reveals a general trend with regard to the sheets of MoS_2_ homogeneously loaded on graphene. The MoS_2_ layers (black stripes) could be visibly identified and appear to be kept in close contact with graphene layers. The selected area electron diffraction (SAED) pattern is shown in the inset of Fig. 1(b). Moreover, distinct electron diffraction patterns from the (100) and (110) planes of graphene and (100), (110) and (118) planes of MoS_2_ also confirmed the simultaneous appearance of both graphene and MoS_2_. The UV–Vis spectra were used to demonstrate that the MoS_2_/graphene nanocomposites had been successfully synthesized. As shown in Fig. 1(c), the spectral peaks of the MoS_2_/graphene nanocomposites (red solid line) at 610 and 670 nm correspond to the two characteristic absorption peaks of MoS_2_ (blue dotted line), whereas, the increase of light absorption at wavelengths shorter than 500 nm is due to the intrinsic absorption from graphene (black dashed line). Raman spectroscopy was carried out to further probe the internal structures of MoS_2_/graphene nanocomposites. The Raman spectrum (Fig. 1(d)) shows the existence of typical E_2g_^1^, A_1g_, 2 LA (M), D and G peaks at 380, 407, 460, 1342 and 1593 cm^−1^, respectively. The in-plane E_2g_^1^ peak results from the opposing vibration of the two S atoms with respect to the Mo atom, whereas the A_1g_ peak is associated with the out-of-plane vibration of only the S atoms in opposite directions^43^. The asymmetric 2 LA (M) peak is associated with the second-order longitudinal acoustic mode at the M point^44^. These results suggest that the structure is relatively undistorted MoS_2_^45^. From the peak distance (27 cm^−1^) between the E_2g_^1^ and A_1g_ modes, the structure of the MoS_2_ in the nanocomposites has been suggested to consist of multiple layers^46^,^47^. The D and G peaks confirmed the presence of graphene within the nanocomposites. All of the above characterizations could effectively illustrate the formation of the MoS_2_/graphene nanocomposites.

### Ultrafast and nonlinear optical properties

**Figure 2:**
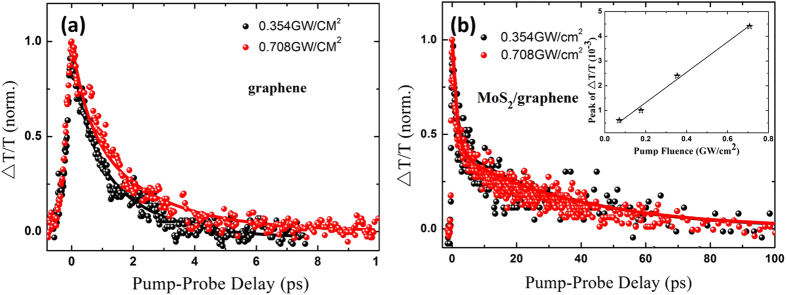
Carrier dynamics of (**a**) graphene and (**b**) MoS_2_/graphene nanocomposites under different pump intensities. Scattered points are experimental results and solid lines are the fitting results. The inset confirms the linear dependence of the pump−probe signal on the pump intensity.

Ultrafast pump-probe and open aperture Z-scan measurements were used to investigate the ultrafast and nonlinear optical properties of the MoS_2_/graphene nanocomposites. The ultrafast carrier dynamics play a critical role in MoS_2_/graphene nanocomposites based optoelectronic applications^48^. The time-dependent transmissivity changes (Δ*T*/*T*) of graphene and MoS_2_/graphene nanocomposites at room temperature are shown in Fig. 2. Here, Δ*T*/*T* is defined as the relative change of the probe transmissivity caused by the pump:


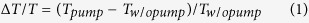


where, 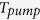 and 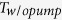 are the transmissions of the probe with and without the presence of the pump, respectively. As a reference, the pump-probe measurement was first performed on graphene. As shown in Fig. 2(a), the ultrafast carrier dynamics is consistent with previous reports^9^,^10^,^11^. The positive Δ*T*/*T* is ascribed to the Pauli blocking induced by excitation based on hot carrier dynamics. Due to the identical wavelengths of the pump and probe pulses, upon the pump excitation, photon-induced carriers or excitons will occupy the probe transition states and reduce the probe photon absorption, leading to a positive Δ*T*. The time-dependent Δ*T*/*T* curve can be successfully fitted to a bi-exponential decay function:


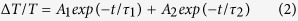


where, 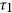 is the decay time with the respective amplitude weights 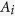
11. All of the fitting results are summarized in Table 1. According to hot carrier dynamics in graphene, the initial fast relaxation time constant 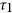 (0.47 ± 0.03 ps/0.51 ± 0.03 ps) corresponds to the coupling between the excited charge carriers and optical phonon mode (carrier-phonon scattering)^9^. The slower constant 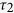 (1.7 ± 0.1 ps/2.6 ± 0.1 ps) is attributed to the cooling time of the thermalized carrier-phonon coupling system and electron–hole recombination^11^, which is dependent on the pump intensity. The order of magnitude of the relaxation times obtained in our experiment corresponds well with previously reported observations^9^,^10^,^11^.

**Figure 3:**
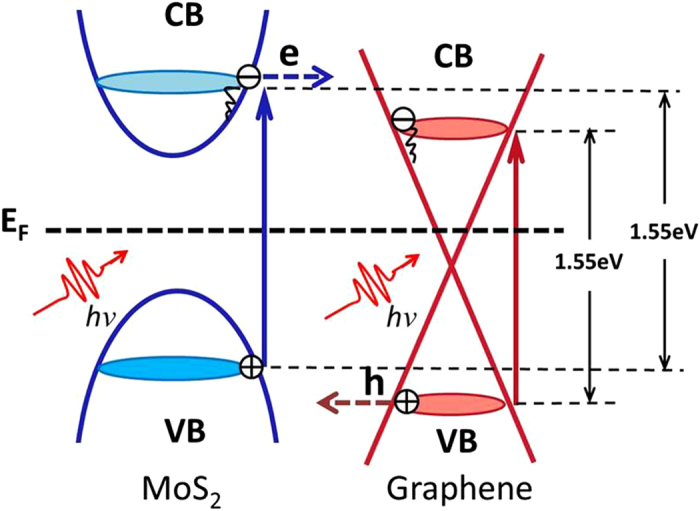
Photo-induced electron transfer from MoS_2_ to graphene upon photon excitation.

A similar positive Δ*T*/*T* was also observed in the MoS_2_/graphene nanocomposites. However, the carrier relaxation time of the nanocomposites is considerably longer than that of graphene (Fig. 2). The fast and slower relaxation time constants are 1.3 ± 0.2 ps/1.4 ± 0.2 ps 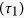 and 36 ± 2 ps/37 ± 2 ps 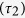, respectively. The carrier dynamics under different pump intensities was investigated to understand the slower relaxation mechanism in the nanocomposites. As shown in Fig. 2(b), there is no relaxation time dependence on the pump power intensity in the nanocomposites. However, the carrier relaxation of graphene is typically dependent on the pump-induced carrier density, higher pump power leads to a slower observed relaxation time^9^. Therefore, carrier–optical phonon interactions in pristine graphene are excluded from the carrier dynamics in the nanocomposites. The inset of Fig. 2(b) shows that the maximum value of the transmissivity change (Δ*T*/*T*) is proportional to the pump power intensity when the intensity is below 0.8 GW/cm^2^, which indicated all these experiments were conducted in the linear absorption range. Therefore, the saturation of optical transitions is not likely to be the reason for the intensity-independent slower relaxation time in the MoS_2_/graphene nanocomposites. Thus, the interaction between graphene and MoS_2_ is considered to play a significant role in their relaxation dynamics mechanism, as illustrated in Fig. 3.

The utility of graphene as a perfect electron acceptor has been proven in many systems^49^,^50^,^51^. In the MoS_2_/graphene nanocomposites with the covalent donor–acceptor structure, the Fermi energy of MoS_2_ (E_F_) is higher than that of graphene. Therefore, MoS_2_ can act as an electron donor through covalently bonding with graphene, thus facilitating the electron transfer from MoS_2_ to graphene. The details are described as follows. Upon photoexcitation with the 1.55 eV (800 nm) pump pulse, electrons can be rapidly transited from the valence band to the conduction band, in both graphene with its gapless band structure^7^ and multilayer MoS_2_ with its indirect gap energy of below 1.48 eV^28^. The excited state electrons of MoS_2_ can be efficiently transferred to graphene, due to the slower excited state electron relaxation (hundred ps) of the pristine MoS_2_^52^,^53^,^54^,^55^. This photon-induced electron transfer process may dominate and further disrupt the carrier relaxation occurring at the graphene, which could be the main cause of the slower delay time observed in the MoS_2_/graphene nanocomposites.

**Figure 4:**
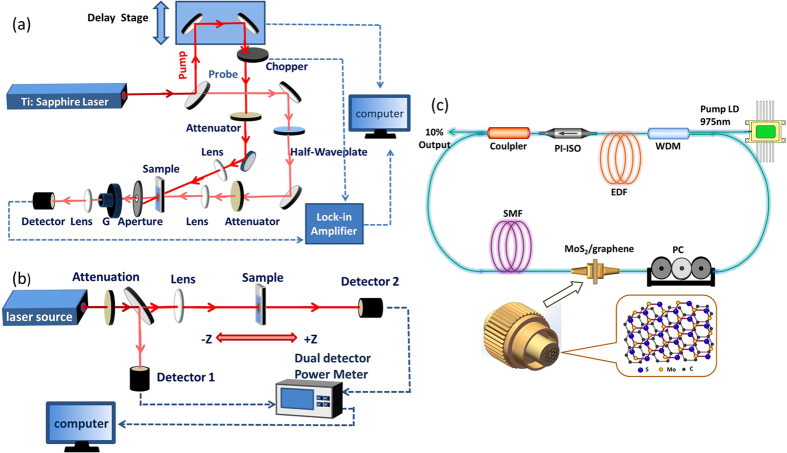
Experimental setup of (**a**) the pump-probe measurement, (**b**) the open aperture Z-scan technique and (c) a schematic of the erbium-doped fiber laser passive Q-switcher and mode-locker with the MoS_2_/graphene nanocomposites-based SA. WDM (wavelength division multiplexer), EDF (erbium-doped fiber), PI-ISO (polarisation-independent isolator), SMF (single-mode fiber), PC (polarisation controller), and MoS_2_/graphene nanocomposites based saturable absorber. (All of the images were created by the authors).

**Figure 5:**
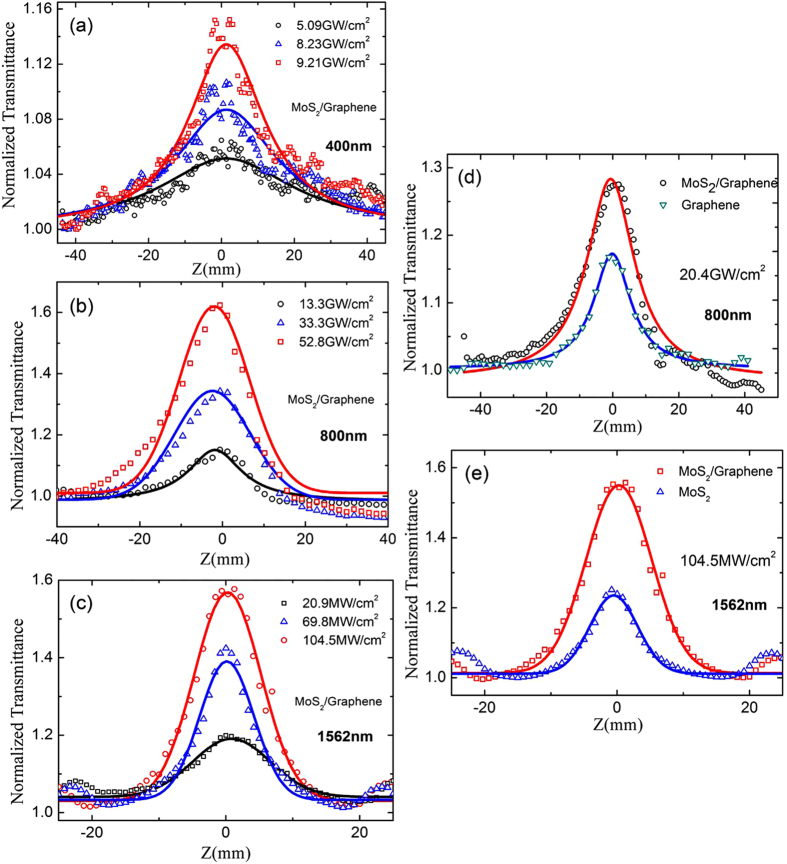
Open-aperture Z-scan measurement of the MoS_2_/graphene nanocomposites at different input influences at 400 nm (**a**), 800 nm (**b**) and 1562 nm (**c**). (**d**) Open-aperture Z-scan curves of the MoS_2_/graphene nanocomposites and graphene at the same input influences of 20.4 GW/cm^2^ at 800 nm. (**e**) MoS_2_/graphene nanocomposites and MoS_2_ at the input influences of 104.5 mW/cm^2^ at 1562 nm.

The open-aperture Z-scan measurements were also made under variable optical powers from the visible to the near-infrared broadband frequency range. A piece of β-BaB_2_O_4_ (BBO) crystal was used to double the frequency of the incident femto-second laser pulse at 800 nm through Second Harmonic Generation (SHG), allowing for the generation of laser pulses at 400 nm. In Fig. 4(b), the beam waist radii of the focused Gaussian beams were approximately 5 and 25 μm, in the open-aperture Z-scan measurements at 800 and 400 nm, respectively. Figure 5(a,b) show the typical saturable absorption curves of the intensity-dependent open-aperture Z-scan measurements, when the MoS_2_/graphene nanocomposites were moved along the beam focus, indicating the clear dependence of the transmittance peak on the input intensity.

To verify whether MoS_2_/graphene nanocomposites show saturable absorption at near-infrared wavelengths, a home-made Erbium-doped femtosecond fiber laser (central wavelength: 1562.6 nm, repetition rate: 20.13 MHz and pulse duration: 565 fs) was also used as an excitation source. After being focused by a 10 cm focal-length lens, the laser beam was measured to be approximately 10 μm in diameter at the focal point. The saturable absorption curves were identified at different input intensities, as shown in Fig. 5(c).

Figure 5(d) shows the saturable absorption comparison of the open-aperture Z-scan curves for MoS_2_/graphene nanocomposites and graphene under the on-axis peak intensity *I*_0_ of 20.4 GW/cm^2^ at 800 nm. Figure 5(e) shows the results for the MoS_2_/graphene nanocomposites and MoS_2_ under 104.5 mW/cm^2^ at 1562 nm. In contrast with graphene and MoS_2_, the MoS_2_/graphene nanocomposites exhibit larger transmittance values with the same input intensity toward the focus, indicating an enhanced light-matter interaction compared to graphene or MoS_2_.

The Z-scan measurement results are fitted by the equation^25^:


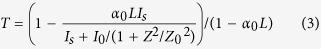


where, *T* is the transmittance, *L* is the sample length, 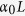 is the modulation depth, 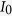 is the peak intensity, 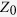 is the diffraction length of the beam and 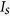 is the saturable intensity.

From these nonlinear optics fitting curves at different wavelengths, the saturable intensity and modulation depth are summarized in Table 2. Our results indicated that MoS_2_/graphene nanocomposites can be considered to be a broadband saturable absorber with an operation regime from the visible to the near-infrared. Meanwhile, compared with graphene and MoS_2_, the MoS_2_/graphene nanocomposites possess equivalent saturable intensity along with larger modulation depth. The larger modulation depth indicates that the MoS_2_/graphene nanocomposites may be a promising candidate for passive laser Q-switcher and mode-locker for the generation of ultrashort pulse.

### Q-switched and mode-locked fiber laser

**Figure 6:**
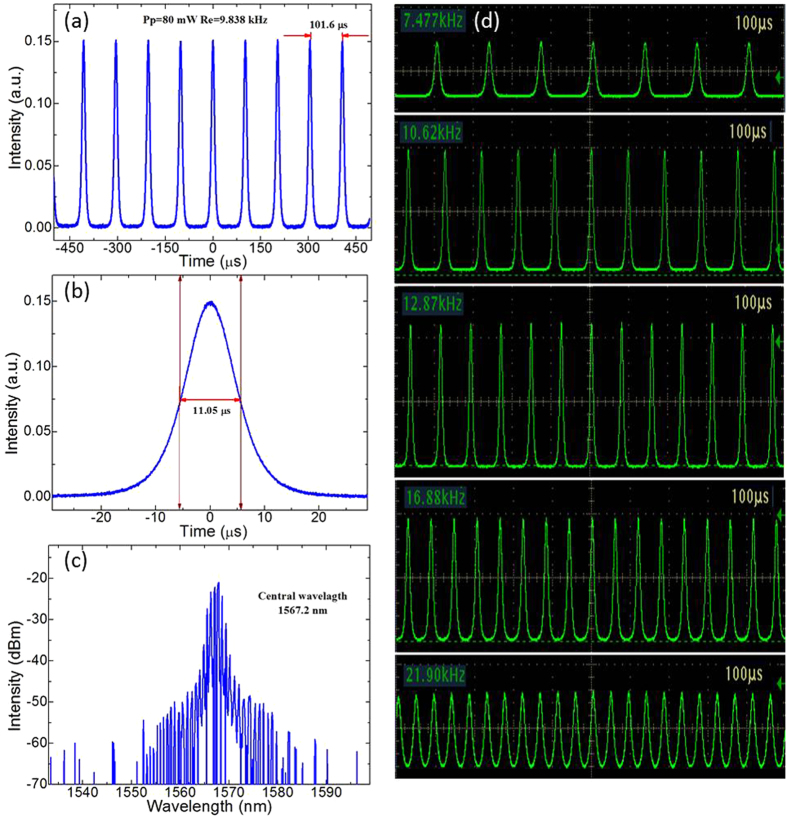
(**a**) Typical Q-switching pulse train, (**b**) single pulse profile and (**c**) output spectrum of the fiber laser obtained at a pump power of 80 mW. (**d**) Evolution of Q-switching pulse trains with increasing pump power.

The broadband saturable absorption of the MoS_2_/graphene nanocomposites has allowed fabrication of a new type of 2D nanocomposites-based optical fiber saturable absorber device, as shown in Fig. 4(c). Once the pump power exceeded a threshold of 50 mW, stable Q-switching operation self-started immediately without any adjustment of the polarization controller (PC). The typical Q-switching characteristics at a pump power (Pp) of 80 mW are detailed in Fig. 6. Figure 6(a) shows the pulse train with a repetition rate (Rep) of 9.838 kHz, in which the uniform intensity distribution indicates the good stability of this output pulse train. To further investigate the pulse details, we measured the corresponding single pulse profile with a narrower sweep span as shown in Fig. 6(b). The pulse had a full width at half maximum (FWHM) of 11.05 μs without any random modulations on the top of the pulse, suggesting the fine suppression of Q-switching instability. The corresponding output spectrum is shown in Fig. 6(c). Our Q-switched fiber laser started oscillations at a wavelength of 1567.2 nm. We have also investigated the evolution of Q-switching pulse trains with increasing the pump power, as shown in Fig. 6(d). The results illustrate that the pulse interval gradually deceases with an increase in pump power, which is a typical characteristic of the Q-switching state. Moreover, the pulse trains always maintain a uniform intensity distribution. These experimental results demonstrate the successful performance of our fiber laser.

**Figure 7:**
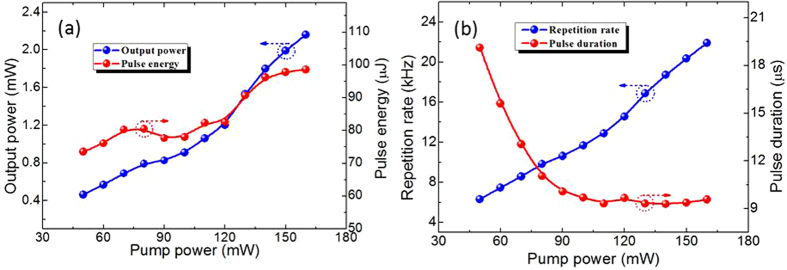
(**a**) Output average power and pulse energy. (**b**) Pulse repetition rate and duration versus incident pump power.

Furthermore, we have studied the evolution of the Q-switching parameters with an increase in pump power. The details are given in Fig. 7. The initial Q-switching pulses had a pulse width of 19.12 μs, a repetition rate of 6.312 kHz and an output power of 464 μW, corresponding to pulse energy of 73.5 nJ. As the pump power is increased, the pulse duration decreased nonlinearly, finally reaching approximately zero. However, the output average power and repetition rate increased nearly linearly. Q-switching operation could be maintained until the pump power exceeded 165 mW, with a maximum output power of 2.16 mW, a pulse repetition rate of 21.9 kHz and corresponding pulse energy of 98.6 nJ. The minimum pulse duration is approximately 9.31 μs.

**Figure 8:**
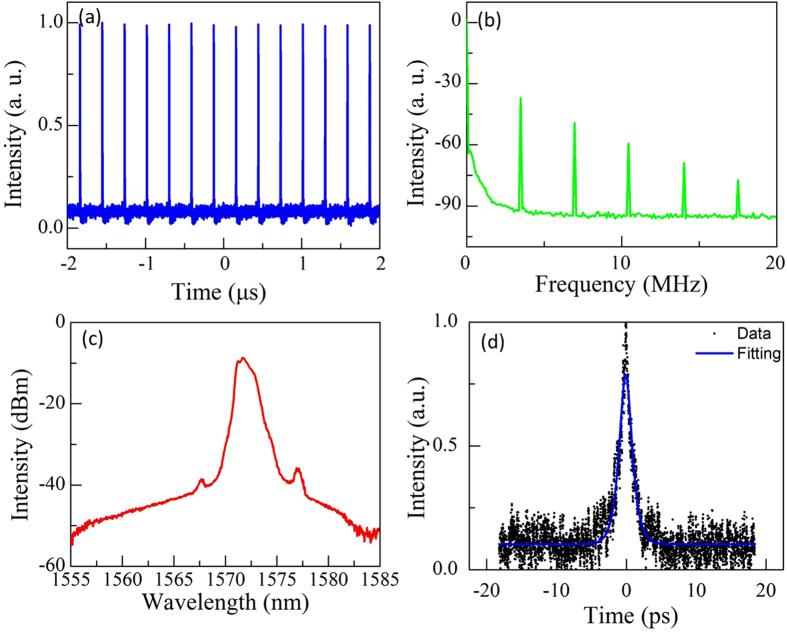
Typical mode-locked soliton pulse at a pump power of 80 mW. (**a**) Pulse train. (**b**) RF spectrum. (**c**) Optical spectrum. (**d**) Autocorrelation trace.

Aside from the passive Q-switching operation, we were also able to demonstrate the passive mode-locking operation with another relatively thin MoS_2_/graphene nanocomposites-based SA. Mode-locking started once the pump power exceeded a threshold of 70 mW. Figure 8 summarizes the characteristics of the mode-locked pulses at a pump power of 80 mW. Figure 8(a) shows its pulse train with a uniform intensity distribution. The repetition rate of 3.47 MHz revealed by this data precisely matches the time interval of 285.2 ns. Figure 8(b) presents the corresponding RF spectrum with a span of 20 MHz and a resolution bandwidth of 100 Hz. The repetition rate of the soliton pulses is 3.47 MHz, corresponding to the cavity length of ~57.49 m. The signal-to-noise ratio (SNR) of ~53.7 dB is an indication of good mode-locking stability. Figure 8(c) shows that the realized laser output started oscillating at a wavelength of 1571.8 nm with 3-dB bandwidth of 1.5 nm. As a typical characteristic of conventional soliton pulses, the Kelly sidebands have been shown at both sides of the spectrum. A corresponding auto-correlation trace with a full width at half maximum (FWHM) of 3.67 ps is shown in Fig. 8(d). The actual pulse duration is 2.2 ps with an assumed sech^2^ pulse profile. The time-bandwidth product (TBP) of the pulses is ~0.405, indicating that the output pulses are slightly chirped. The above-mentioned experimental results also support the excellent performance of our fiber laser. Multiple soliton operations were observed when the pump power was increased to 120 mW.

## Discussion

In the pump-probe measurements, the MoS_2_/graphene composites exhibit a fast delay time constant in the range of 1.3–1.4 ps and a slower constant in the 36–37 ps range. According to the slower delay time than that of graphene, we consider that graphene and MoS_2_ formed a covalent donor–acceptor structure. Indeed, during the excitation of the MoS_2_/graphene nanocomposites, there is an energy transfer from the excited states of MoS_2_ to graphene followed by the non-radiative relaxation of graphene’s excited state carriers to the ground state. Due to the excited state electron relaxation time of graphene and MoS_2_, we speculate that the carriers reach the ground state at picosecond relaxation times. Thus, the strong absorption of the nanocomposites and the immediate energy transfer from MoS_2_ to graphene could influence the excited state absorption cross section of graphene and further enhance the light-induced bleaching effect. Therefore, their covalent donor–acceptor structure is considered to be important in the mechanisms of the enhancement of nonlinear saturable absorption in the MoS_2_/graphene nanocomposites.

According to the open aperture Z-scan measurements at various wavelengths from the visible to the near-infrared range, the MoS_2_/graphene nanocomposites are experimentally found to possess the enhanced and broadband saturable absorption. Taking advantage of their excellent saturable absorption property, a novel optical saturable absorber device based on MoS_2_/graphene nanocomposites was fabricated. The photonics application for ultra-short pulsed laser operation at the telecommunication band (1.5 μm) was also demonstrated. Our work provides a new type of tunable 2D photonics materials by combining the optical advantages of different 2D materials. We foresee that their widespread applications for ultrashort pulse generation or optical communications will be increasingly compelling and promising.

## Methods

### Preparation of MoS_2_/graphene nanocomposites

As a precursor, graphene oxide (GO) was synthesized from graphite powder (>99.8%, Sinopharm Chemical Reagent Co. Ltd) by the modified Hummer’s method by using a mixture of H_2_SO_4_, NaNO_3_, and KMnO_4_^56^. The MoS_2_/graphene nanocomposites were obtained via a hydrothermal method^57^. 30 mL of GO brown colloidal dispersion (1 mg/mL) was prepared by ultrasonication for more than 1 h. Subsequently, 20 mL of 70 mmol cationic surfactant (dodecyltrimethylammonium bromide) solution was added to the resulting GO aqueous dispersion and stirred at room temperature for 12 h to form a mixture. The prepared solution was mixed with 20 mL of a mixed solution of 1.5 mmol Na_2_MoO_4_·2H_2_O and 7.5 mmol L-cysteine (Lcys) with continuous stirring for 30 min. The suspension was then transferred into a 100 mL Teflon-lined stainless steel autoclave and tightly sealed at 240 °C for 24 h. Finally, after being washed several times with deionized water and ethanol, the as-synthesized product was collected by centrifugation, and then naturally cooled and dried in the vacuum oven at 80 °C for 12 h. The obtained black solid product was then annealed at 800 °C for 2 hours with 10% hydrogen in nitrogen flow at 200 sccm to yield the solid MoS_2_/graphene nanocomposites.

In our experimental measurements, MoS_2_ is a pristine nanoflake solution purchased from Graphene Supermarket. The graphene from Nanjing SCF Nanotech was dispersed in N-methyl-2-pyrrolidone (NMP) at a concentration of 2 mg/mL and the solid MoS_2_/graphene nanocomposites were dispersed in NMP at the same concentration.

### Experimental setups of the pump-probe and open aperture Z-scan measurements

A full Ti: sapphire amplified laser system (Libra-S, Coherent) with pulse repetition rate of 1 kHz, a center wavelength at 800 nm, and a pulse width of approximately 100 fs was used as the excitation source. The experimental setup of the pump-probe measurement is shown in Fig. 4(a). The output was split into two beams: the intense portion as the pump beam to generate the photo-induced carriers, and the other weak portion as the probe beam to monitor the changes of the transmittivity of the samples at various delays of the probe pulses relative to the pump pulses. The delay time between the two pulses was controlled by a translation stage (Zolix, KSA300-12-X). The pump and probe beams were focused to spot sizes with diameters of approximately 600 and 100 μm, respectively. The pump and probe beams are orthogonally polarized by placing a half-wave plate in the probe arm and rotating its polarization by 90°. A Glan-Taylor polarizer (G) was placed in front of the detector and set at the same polarization direction as the probe beam to minimize the scattered pump noise. The pump beam was modulated at a frequency of 290 Hz by an optical chopper. The transmittance changes of the probe pulses were measured with a photoelectric detector connected to a lock-in amplifier (SR850).

The Z-scan experimental setup is shown in Fig. 4(b). Different types of laser setups were employed as the laser excitation source to fully characterize the broadband nonlinear optical response of the MoS_2_/graphene nanocomposites. The incident laser pulses were also divided into two beams: the reflected beam was used as the reference, and the transmitted beam was focused by a lens as the excitation. The beam waist radius of the focused Gaussian beam was determined by a CCD camera. The pulse powers of the transmitted and reference beams were simultaneously measured by a dual-channel power meter (Newport).

Both pump-probe and open-aperture Z-scan measurements were performed on graphene, MoS_2_ and the MoS_2_/graphene nanocomposites dispersion prepared in 1 mm-thick quartz cells. A comparison experiment on the pure solvents (NMP) was carried out, and no clear signal was observed for either pump-probe or Z-scan experiments. Meanwhile, the reproducibility of the transmission curves was tested by cycling the laser intensity up and down to further ensure that the detected materials were free of optical damage.

### MoS_2_/graphene nanocomposites-based SA fabrication

The MoS_2_/graphene nanocomposites dispersion in NMP was drop-casted twice directly onto a fiber end-facet, which was then inserted in a standard FC/PC fiber connector for Q-switching operation. To achieve mode-locking operation, we drop-casted the dispersion only once onto another fiber end-facet so that the obtained SA was relatively thin. Allowing the fiber end-facets to dry at room temperature for approximately 10 h allowed us to obtain an even self-assembly of the MoS_2_/graphene nanocomposites molecules onto the fiber end-facets, as shown in Fig. 4(c). The MoS_2_/graphene nanocomposites-based SA device was successfully constructed for the fiber laser application by connecting the MoS_2_/graphene nanocomposites-on-fiber component with another clean, dry FC/PC fiber connector.

### Schematic setup of the passive Q-switched and mode-locked fiber laser

Figure 4(c) illustrates the schematic setup of the passive Q-switched and mode-locked fiber laser. Through 980/1550 nm wavelength division multiplexer (WDM), the pumping light from a 975 laser diode (LD, 500 mW) was coupled into a piece of 0.9 m highly-doped erbium-doped fiber (EDF, LIEKKI Er 80-8/125) as gain medium. A 10% coupler was used as the output port. Besides the gain fiber, all the remaining fibers used in the cavity were all standard single mode fiber (SMF, SMF-28) with total cavity length of approximately 57.55 m. The Polarization-Independent Isolator (PII-ISO) and Polarization Controller (PC) were used to maintain the unidirectional operation and optimize the performance of our fiber laser, respectively.

## Additional Information

**How to cite this article**: Jiang, Y. *et al.* Broadband and enhanced nonlinear optical response of MoS_2_/graphene nanocomposites for ultrafast photonics applications. *Sci. Rep.* **5**, 16372; doi: 10.1038/srep16372 (2015).

## Figures and Tables

**Table 1:** Fitting parameters for the experimental data in Fig. 2.

Dispersions	Power (*GW/cm*^*2*^)	*A*_*1*_	*A*_*2*_	*τ*_*1*_*(ps)*	*τ*_*2*_ (*ps*)
graphene	0.354	0.409	0.591	0.47 ± 0.03	1.7 ± 0.1
graphene	0.708	0.389	0.611	0.51 ± 0.03	2.6 ± 0.1
MoS_2_/graphene	0.354	0.564	0.436	1.3 ± 0.2	36 ± 2
MoS_2_/graphene	0.708	0.593	0.407	1.4 ± 0.2	37 ± 2

**Table 2:** NLO parameters of MoS_2_/graphene composites and graphene fitting from the experimental data analysis in Fig. 4: *I*_*s*_: saturable intensity; *a*_0_*L*: modulation depth.

Material	*λ*(*nm*)	Duration time (*fs*)	*I*_*S*_	*α*_*0*_*L*
MoS_2_/graphene	400	100	1.427 GW/cm^2^	13.09%
MoS_2_/graphene	800	100	2.02 GW/cm^2^	48%
graphene	800	100	2.5 GW/cm^2^	16.34%
MoS_2_/graphene	1562.6	565	2.44 mW/cm^2^	38.3%
MoS_2_	1562.6	565	2.23 mW/cm^2^	20.46%

## References

[b1] Wang Q. H. *et al.* Electronics and optoelectronics of two-dimensional transition metal dichalcogenides. Nature Nanotechnol. 7, 699–712 (2012). 23132225 10.1038/nnano.2012.193

[b2] Zhang Y., Tan Y. W., Stormer H. L. & Kim P. Experimental observation of the quantum Hall effect and Berry’s phase in graphene. Nature 438, 201–204 (2005). 16281031 10.1038/nature04235

[b3] Novoselov K. S. A. *et al.* Two-dimensional gas of massless Dirac fermions in graphene. Nature 438, 197–200 (2005). 16281030 10.1038/nature04233

[b4] Xu H. *et al.* Batch-fabricated high-performance graphene Hall elements. Sci. Rep. 3, 1207 (2013). 23383375 10.1038/srep01207 PMC3563039

[b5] Mak K. F., Lui C. H., Shan J. & Heinz T. F. Observation of an electric-field-induced band gap in bilayer graphene by infrared spectroscopy. Phys. Rev. Lett. 102, 256405 (2009). 19659105 10.1103/PhysRevLett.102.256405

[b6] Wang J. *et al.* Broadband nonlinear optical response of graphene dispersions. Adv. Mater. 21, 2430–2435 (2009). 10.1364/OE.21.016486 23938499

[b7] Nikolaenko A. E. *et al.* Nonlinear graphene metamaterial. Appl. Phys. Lett. 100, 181109 (2012).

[b8] Ishida Y. *et al.* Non-thermal hot electrons ultrafastly generating hot optical phonons in graphite. Sci. Rep. 1, 64 (2011). 22355583 10.1038/srep00064 PMC3216551

[b9] Huang L. *et al.* Ultrafast transient absorption microscopy studies of carrier dynamics in epitaxial graphene. Nano lett. 10, 1308–1313 (2010). 20210348 10.1021/nl904106t

[b10] Dawlaty J. M. *et al.* Measurement of ultrafast carrier dynamics in epitaxial graphene. Appl. Phys. Lett. 92, 042116 (2008).

[b11] Chen K. *et al.* Diversity of ultrafast hot-carrier-induced dynamics and striking sub-femtosecond hot-carrier scattering times in graphene. Carbon 72, 402–409 (2014).

[b12] Shmeliov A. *et al.* Unusual Stacking Variations in Liquid-Phase Exfoliated Transition Metal Dichalcogenides. ACS nano 8, 3690–3699 (2014). 24588696 10.1021/nn5003387

[b13] Li H. M. *et al.* Metal-Semiconductor Barrier Modulation for High Photoresponse in Transition Metal Dichalcogenide Field Effect Transistors. Sci. Rep. 4, 4041 (2014). 24509565 10.1038/srep04041 PMC3918928

[b14] Zeng H. *et al.* Optical signature of symmetry variations and spin-valley coupling in atomically thin tungsten dichalcogenides. Sci. Rep. 3, 1608 (2013). 23575911 10.1038/srep01608 PMC3622914

[b15] Coleman J. N. *et al.* Two-dimensional nanosheets produced by liquid exfoliation of layered materials. Science 331, 568–571 (2011). 21292974 10.1126/science.1194975

[b16] Radisavljevic B. *et al.* Single-layer MoS2 transistors. Nature Nanotechnol. 6, 147–150 (2011). 21278752 10.1038/nnano.2010.279

[b17] Cho K. *et al.* Electric Stress-Induced Threshold Voltage Instability of Multilayer MoS_2_ Field Effect Transistors. ACS nano 7, 7751–7758 (2013). 23924186 10.1021/nn402348r

[b18] Ghatak S., Pal A. N. & Ghosh A. Nature of electronic states in atomically thin MoS_2_ field-effect transistors. Acs Nano 5, 7707–7712 (2011). 21902203 10.1021/nn202852j

[b19] SungáLee H., JoonáChoi H. & KyuáPark M. Nanosheet thickness-modulated MoS_2_ dielectric property evidenced by field-effect transistor performance. Nanoscale 5, 548–551 (2013). 23233087 10.1039/c2nr33443g

[b20] Lopez-Sanchez O. *et al.* Ultrasensitive photodetectors based on monolayer MoS_2_. Nature Nanotechnol. 8, 497–501 (2013). 23748194 10.1038/nnano.2013.100

[b21] Choi M. S. *et al.* Controlled charge trapping by molybdenum disulphide and graphene in ultrathin heterostructured memory devices. Nature commun. 4, 1624 (2013). 23535645 10.1038/ncomms2652

[b22] Wang H. *et al.* Integrated circuits based on bilayer MoS_2_ transistors. Nano lett. 12, 4674–4680 (2012). 22862813 10.1021/nl302015v

[b23] Yu W. J. *et al.* Vertically stacked multi-heterostructures of layered materials for logic transistors and complementary inverters. Nature mater. 12, 246–252 (2013). 23241535 10.1038/nmat3518 PMC4249642

[b24] Du J. *et al.* Ytterbium-doped fiber laser passively mode locked by few-layer Molybdenum Disulfide (MoS_2_) saturable absorber functioned with evanescent field interaction. Sci. Rep. 4, 6346 (2014). 25213108 10.1038/srep06346 PMC4161963

[b25] Zhang H. *et al.* Molybdenum disulfide (MoS_2_) as a broadband saturable absorber for ultra-fast photonics. Opt. express 22, 7249–7260 (2014). 24664073 10.1364/OE.22.007249

[b26] Tsai D. S. *et al.* Few-layer MoS_2_ with high broadband photogain and fast optical switching for use in harsh environments. Acs Nano 7, 3905–3911 (2013). 23590667 10.1021/nn305301b

[b27] Lee H. S. *et al.* MoS_2_ nanosheet phototransistors with thickness-modulated optical energy gap. Nano lett. 12, 3695–3700 (2012). 22681413 10.1021/nl301485q

[b28] Mak K. F. *et al.* Atomically thin MoS_2_: a new direct-gap semiconductor. Phys. Rev. Lett. 105, 136805 (2010). 21230799 10.1103/PhysRevLett.105.136805

[b29] Britnell L. *et al.* Strong light-matter interactions in heterostructures of atomically thin films. Scienc 340, 1311–1314 (2013). 10.1126/science.1235547 23641062

[b30] Britnell L. *et al.* Field-effect tunneling transistor based on vertical graphene heterostructures. Science 335, 947–950 (2012). 22300848 10.1126/science.1218461

[b31] Loomis J. *et al.* Graphene/elastomer composite-based photo-thermal nanopositioners. Sci. Rep. 3, 1900 (2013). 23712601 10.1038/srep01900 PMC3664893

[b32] Chang K. & Chen W. L-cysteine-assisted synthesis of layered MoS_2_/graphene composites with excellent electrochemical performances for lithium ion batteries. ACS Nano 5, 4720–4728 (2011). 21574610 10.1021/nn200659w

[b33] Xiao J. *et al.* Electrochemically induced high capacity displacement reaction of PEO/MoS_2_/graphene nanocomposites with lithium. Adv. Funct. Mater. 21, 2840–2846 (2011).

[b34] Liu C. J. *et al.* Facile synthesis of MoS_2_/graphene nanocomposite with high catalytic activity toward triiodide reduction in dye-sensitized solar cells. J. Materials Chemistry 22, 21057–21064 (2012).

[b35] Zhou X. *et al.* Facile synthesis and electrochemical properties of two dimensional layered MoS_2_/graphene composite for reversible lithium storage. J. Power Sources 251, 264–268 (2014).

[b36] Huang K. J., Wang L., Li J. & Liu Y. M. Electrochemical sensing based on layered MoS_2_–graphene composites. Sens. Actuators, B: Chemical 178, 671–677 (2013).

[b37] Xiang Q., Yu J. & Jaroniec M. Synergetic effect of MoS_2_ and graphene as cocatalysts for enhanced photocatalytic H_2_ production activity of TiO_2_ nanoparticles. J. Am. Chem. Soc. 134, 6575–6578 (2012). 22458309 10.1021/ja302846n

[b38] Zhao C. *et al.* Wavelength-tunable picosecond soliton fiber laser with Topological Insulator: Bi_2_Se_3_ as a mode locker. Opt. express 20, 27888–27895 (2012). 23262733 10.1364/OE.20.027888

[b39] Zhao C. *et al.* Ultra-short pulse generation by a topological insulator based saturable absorber. Appl. Phys. Lett. 101, 211106 (2012).

[b40] Sotor J., Sobon G., Grodecki K. & Abramski K. M. Mode-locked erbium-doped fiber laser based on evanescent field interaction with Sb_2_Te_3_ topological insulator. Appl. Phys. Lett. 104, 251112 (2014).

[b41] Sotor J. *et al.* Mode-locking in Er-doped fiber laser based on mechanically exfoliated Sb_2_Te_3_ saturable absorber. Opt. Mater. Express. 4, 1–6 (2014).

[b42] Khazaeizhad R. *et al.* Mode-locking of Er-doped fiber laser using a multilayer MoS_2_ thin film as a saturable absorber in both anomalous and normal dispersion regimes. Opt. express 22, 23732–23742 (2014). 25321840 10.1364/OE.22.023732

[b43] Bertrand P. A. Surface-phonon dispersion of MoS_2_. Phys. Rev. B 44, 5745 (1991). 10.1103/physrevb.44.5745 9998418

[b44] Windom B. C., Sawyer W. G. & Hahn D. W. A Raman spectroscopic study of MoS_2_ and MoO_3_: applications to tribological systems. Trib. Lett. 42, 301–310 (2011).

[b45] Frey G. L. *et al.* Solution-processed anodes from layer-structure materials for high-efficiency polymer light-emitting diodes. J. Am. Chem. Soc. 125, 5998–6007 (2003). 12733940 10.1021/ja020913o

[b46] Li S. L. *et al.* Quantitative Raman spectrum and reliable thickness identification for atomic layers on insulating substrates. ACS nano 6, 7381–7388 (2012). 22838842 10.1021/nn3025173

[b47] Lee C. *et al.* Anomalous lattice vibrations of single-and few-layer MoS2. ACS nano 4, 2695–2700 (2010). 20392077 10.1021/nn1003937

[b48] Bonaccorso F., Sun Z., Hasan T. & Ferrari A. C. Graphene photonics and optoelectronics. Nature Photonics 4, 611–622 (2010).

[b49] Xu Y. *et al.* A graphene hybrid material covalently functionalized with porphyrin: synthesis and optical limiting property. Adv. Mater. 21, 1275–1279 (2009).

[b50] Zhang W. *et al.* Ultrahigh-Gain Photodetectors Based on Atomically Thin Graphene-MoS_2_ Heterostructures. Sci. Rep. 4, 3826 (2014). 24451916 10.1038/srep03826 PMC3899643

[b51] Rao C. N. R., Sood A. K., Voggu R. & Subrahmanyam K. S. Some novel attributes of graphene. J. Phys. Chem. Lett. 1, 572–580 (2010).

[b52] Wang R. *et al.* Ultrafast and spatially resolved studies of charge carriers in atomically thin molybdenum disulfide. Phys. Rev. B 86, 045406 (2012).

[b53] Wang Q. *et al.* Valley Carrier Dynamics in Monolayer Molybdenum Disulfide from Helicity-Resolved Ultrafast Pump–Probe Spectroscopy. ACS Nano 7, 11087–11093 (2013). 24224951 10.1021/nn405419h

[b54] Shi H. *et al.* Exciton dynamics in suspended monolayer and few-layer MoS_2_ 2D crystals. ACS Nano 7, 1072–1080 (2013). 23273148 10.1021/nn303973r

[b55] Wang R. *et al.* Optical pump-probe studies of carrier dynamics in few-layer MoS_2_. *Preprint arXiv*:1110.6643 (2011).

[b56] Qi X. *et al.* Ultraviolet, visible, and near infrared photoresponse properties of solution processed graphene oxide. Appl. Surf. Sci. 266, 332–336 (2013).

[b57] Ma L. *et al.* Cationic surfactant-assisted hydrothermal synthesis of few-layer molybdenum disulfide/graphene composites: Microstructure and electrochemical lithium storage. J. Power Sources 264, 262–271 (2014).

